# New Operation for the Cure of Varicose Veins

**Published:** 1872-03

**Authors:** 


### New Operation for the Cure of Varicose Veins.

Mr. Stokes has been recently treating varicose veins on a plan which was suggested to him by Sir Dominic Corrigan. It occurred to Sir Dominic that as hemorrhoidal tumours are, as a rule, so successfully treated by the application of strong nitric acid, the application of this acid to varicose veins in other situations would probably be attended with equally good results. In a case which is still under observation in the Richmond Hospital, this plan of treatment has been attended with the happiest results. The patient is a young man aged 21, and was admitted into the hospital on the 15th of last month. He had a varicose tumour, of the size of a small orange, on the inner aspect of the middle third of the right leg. It had existed for seven years. He suffered also from a large varicose ulcer, which existed over the inner ankle of the same leg; and there was also a second tumour, formed of a cluster of varicose veins, in the right groin. Mr. Stokes performed the operation in the following way. Pressure having been made above and below the tumour, the integuments were raised from the tumour, and an incision, by transfixion, was made over the veins. The *fuming* nitric acid was then applied to the external coats af the veins. No pain attended this application. On the following day the contents of the tumour appeared solidified at the base; and the acid was again applied. The process of solidification then went on rapidly, the tumour at the same time decreasing in size. A week after the operation, some coagulated blood appeared at the site of the operation; and the following day a portion of the vein came away. This was followed by a slight local inflammation; which, however, after a few days, quite subsided. The wound was then for some days dressed with tinct. benzoin co. and glycerine; and it rapidly healed, as did also the ulcer; and the large varicose tumour in the groin entirely disappeared.—*British Medical Journal, May* 28, 1871, *p.* 550.—*Braithwaite's Retrospect.*

